# 1138. A Scoping Review of the Role of the Provider in HPV Vaccination Dyads Research, 2012-2022

**DOI:** 10.1093/ofid/ofad500.979

**Published:** 2023-11-27

**Authors:** Alyssa F Clare, Claudia L Gaviria Agudelo, Kimberly Walker

**Affiliations:** University of South Florida Morsani College of Medicine, Tampa, Florida; University of South Florida Morsani College of Medicine, Tampa, Florida; University of South Florida Zimmerman School of Advertising & Mass Communications, Tampa, Florida

## Abstract

**Background:**

Despite the influence of provider recommendation on Human papillomavirus (HPV) vaccine uptake, few studies in the last decade have assessed the provider’s role in impacting parent and adolescent decisions regarding this vaccine. Given the increasing role of shared decision-making in health decisions, we sought to identify studies that assessed the role of the provider in affecting HPV vaccine decisions among parent-adolescent dyads.

**Methods:**

Inclusion criteria: English-written, original studies from 2012-2022 with a sample including a parent and at least one of the parent’s own adolescents, and with a focus on parent-adolescent intentions and decisions about HPV vaccination. Initial retrieval resulted in 256 studies screened, with 14 meeting eligibility, and seven meeting all qualifications for inclusion.

**Results:**

Clinicians were found to be influential in a dyad’s shared decision to vaccinate against HPV, especially when providing a rationale for vaccination (1, 4, 6). The dyad’s intention to speak with a provider was positively correlated with the likelihood of getting vaccinated (r=0.37, p< 0.05) (5). Both dyad members had high confidence in their provider’s ability to answer questions regarding HPV vaccination (3). Each dyad member felt that the HPV vaccine was discussed more than other vaccines (1). One study explored the relationship between provider-obtained information and adolescent risk perceptions. Less knowledge regarding the HPV vaccine was independently associated with females’ perception of less need for safe sexual practices (p< 0.10) (6). Lastly, delay in receiving the final vaccine dose was associated with decreased accessibility for immunization appointments (7).

**Figure:**
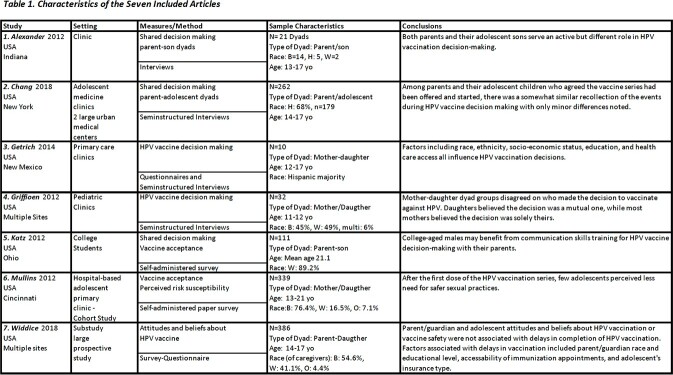

**Conclusion:**

Providers play a significant role in HPV education, as they continue to serve as an important source of information for parents and adolescents. Each dyad member perceives unique benefits and risks to HPV vaccination. When approaching a dyad, providers should attempt to include both the adolescent and the parent in the discussion. Future directions for this research include exploring if addressing dyad members individually improves HPV vaccine uptake.

**Disclosures:**

**All Authors**: No reported disclosures

